# Non-neuronal, slow GABA signalling in the ventrobasal thalamus targets δ-subunit-containing GABA_A_ receptors

**DOI:** 10.1111/j.1460-9568.2011.07645.x

**Published:** 2011-04

**Authors:** Cristina Jiménez-González, Tiina Pirttimaki, David W Cope, H R Parri

**Affiliations:** 1 School of Life and Health Sciences, Aston UniversityBirmingham B4 7ET, UK; 2 Neuroscience Division, School of Biosciences, Cardiff UniversityMuseum Avenue, Cardiff, UK

**Keywords:** astrocyte, glia–neuron interaction, mouse, rat, somatosensory system

## Abstract

The rodent ventrobasal (VB) thalamus contains a relatively uniform population of thalamocortical (TC) neurons that receive glutamatergic input from the vibrissae and the somatosensory cortex, and inhibitory input from the nucleus reticularis thalami (nRT). In this study we describe γ-aminobutyric acid (GABA)_A_ receptor-dependent slow outward currents (SOCs) in TC neurons that are distinct from fast inhibitory postsynaptic currents (IPSCs) and tonic currents. SOCs occurred spontaneously or could be evoked by hypo-osmotic stimulus, and were not blocked by tetrodotoxin, removal of extracellular Ca^2+^ or bafilomycin A1, indicating a non-synaptic, non-vesicular GABA origin. SOCs were more common in TC neurons of the VB compared with the dorsal lateral geniculate nucleus, and were rarely observed in nRT neurons, whilst SOC frequency in the VB increased with age. Application of THIP, a selective agonist at δ-subunit-containing GABA_A_ receptors, occluded SOCs, whereas the benzodiazepine site inverse agonist β-CCB had no effect, but did inhibit spontaneous and evoked IPSCs. In addition, the occurrence of SOCs was reduced in mice lacking the δ-subunit, and their kinetics were also altered. The anti-epileptic drug vigabatrin increased SOC frequency in a time-dependent manner, but this effect was not due to reversal of GABA transporters. Together, these data indicate that SOCs in TC neurons arise from astrocytic GABA release, and are mediated by δ-subunit-containing GABA_A_ receptors. Furthermore, these findings suggest that the therapeutic action of vigabatrin may occur through the augmentation of this astrocyte–neuron interaction, and highlight the importance of glial cells in CNS (patho) physiology.

## Introduction

γ-Aminobutyric acid (GABA)ergic signalling in the thalamus is central to controlling the relay of sensory information to the cortex, and in shaping behavioural state-dependent (patho)physiological cortico-thalamocortical (TC) oscillations (Huguenard & McCormick, 2007). The rodent somatosensory ventrobasal (VB) thalamus contains a relatively uniform population of glutamatergic TC neurons, and unlike most thalamic nuclei is devoid of GABAergic interneurons (Barbaresi *et al.*, 1986; Harris & Hendrickson, 1987). Instead, inhibitory input originates from the nucleus reticularis thalami (nRT), which contains only GABAergic neurons (Spreafico *et al.*, 1991). TC neurons of the VB exhibit two types of GABA_A_ receptor-mediated inhibition: classical fast inhibitory postsynaptic currents (IPSCs), generated by synaptic receptors; and a persistent or tonic current generated by a population of extrasynaptic receptors that are activated by ambient GABA (Brown *et al.*, 2002; Belelli *et al.*, 2005; Cope *et al.*, 2005, Cope *et al.*, 2009; Jia *et al.*, 2005; Chandra *et al.*, 2006; Peden *et al.*, 2008). Importantly, different receptor subtypes underlie the two types of inhibition, synaptic receptors contain α1β2γ2-subunits, whereas α4β2δ-subunits comprise extrasynaptic receptors (Jia *et al.*, 2005; Herd *et al.*, 2009). Indeed, the δ-subunit is located exclusively extrasynaptically and conveys properties on these receptors ideally suited to the generation of tonic currents, particularly slow desensitization and a high affinity for GABA (Saxena & Macdonald, 1994). Despite this high affinity, GABA itself is only a partial agonist at δ-subunit-containing receptors, compared with the hypnotic THIP (Gaboxadol), which is a full, selective agonist at these receptors (Brown *et al.*, 2002; Ebert *et al.*, 2006; Storustovu & Ebert, 2006). Given the molecular, pharmacological and anatomical distinctions between synaptic and extrasynaptic GABA_A_ receptors, they most likely subserve different functions in thalamic (patho)physiological processes (e.g. Cope *et al.*, 2009). Astrocytes are already known to play pivotal roles in the regulation of GABAergic signalling in the somatosensory thalamus as they are the only cell type in this rodent nucleus to express GABA uptake transporters (De Biasi *et al.*, 1998). They therefore form a crucial link in the GABA–glutamine pathway with their uptake of GABA and its subsequent catabolism to glutamine, which is then supplied to nRT afferent presynaptic terminals.

Despite substantial interest in the identity of GABA targets in the thalamus, the sources of GABA that activate GABA_A_ receptors in TC neurons of the VB have not been thoroughly explored. Whilst it is clear that synaptic receptors are directly activated by the vesicular of release of GABA from nRT terminals, whether GABA spillover from these terminals is the sole source of ambient GABA for extrasynaptic receptors has yet to be determined. Non-synaptic sources of GABA, including the reversal of GABA transporters (GATs; Wu *et al.*, 2001, 2003, 2007), have been implicated in other brain regions and, in the olfactory bulb, astrocytes release GABA, probably via a volume-regulated anion channel mechanism (Kozlov *et al.*, 2006). In the VB thalamus, astrocytes can also release gliotransmitters, most notably glutamate that can generate slow inward currents (SICs) in TC neurons and modulate their function (Parri *et al.*, 2001). However, the possibility that astrocytes are a source of GABA in the thalamus has not been evaluated. In this study, we describe spontaneous, transient, non-synaptic slow outward currents (SOC) in TC neurons that are generated by the release of GABA from astrocytes. Despite being transient, SOC are not mediated by synaptic receptors but by δ-subunit-containing extrasynaptic GABA_A_ receptors, and are upregulated by the anti-epileptic vigabatrin.

## Materials and methods

All procedures were carried out in accordance with the UK Animals (Scientific Procedures) Act 1986 and associated procedures.

### Slice preparation

Experiments were performed on postnatal day (P)10–23 for male Wistar rats, and P27–40 for male and female δ-subunit knockout mice (δ KO) and wild-type (WT) littermates. δ KO and WT mice were genotyped as described previously (Cope *et al.*, 2009). Horizontal slices (300–350 μm) of VB thalamus were prepared as described previously (Parri *et al.*, 2001) in ice-cold, continuously oxygenated (95% O_2_: 5% CO_2_) modified artificial cerebrospinal fluid (ACSF) of composition (in mm): NaCl, 126; NaHCO_3_, 26; KCl, 1; KH_2_PO_4_, 1.25; MgSO_4_, 5; CaCl_2_, 1; glucose, 10. Slices were then maintained at room temperature (20 – 24°C) in this solution for a recovery period of 1 h before experimental use.

### Solutions

The standard, continuously oxygenated (95% O_2_: 5% CO_2_) ACSF used in this study contained (in mm): NaCl, 126; NaHCO_3_, 24; KCl, 2.5; KH_2_PO_4_, 1.25; MgSO_4_, 1; CaCl_2_, 2; glucose, 10; unless otherwise stated. Hypo-osmotic stimulus was a 20% decrease in ACSF osmolarity. For experiments where glutamatergic SICs were recorded, Mg^2+^ was omitted to enhance detection (Parri *et al.*, 2001). Tetrodotoxin (TTX) was included in ACSF and hypo-osmotic (h)ACSF in all experiments, except where IPSCs were recorded, and current-clamp experiments. Chemicals for ASCF preparation were obtained from Sigma (St Louis, MO, USA). Pharmacological compounds were included in the ACSF as stated in the text. In the case of Bafilomycin incubation, slices were pretreated in storage ACSF containing 45 μm Bafilomycin A1 for 1–2 h prior to recording. Drugs were obtained from the following sources: (±)-γ-vinyl GABA (vigabatrin), 4,5,6,7-tetrahydroisoxazolo[5,4-c]-pyridin-3-ol (THIP), (5*S*,10*R*)-(+)-5-methyl-10,11-dihydro-5*H*-dibenzo[a,d] cyclohepten-5,10-imine maleate (MK801), (2*S*)-2-amino-2-[(1*S*,2*S*)-2-carboxycycloprop-1-yl]-3-(xanth-9-yl) propanoic acid (LY341495), d-(−)-2-amino-5-phosphonopentanoic acid (D-AP5), bafilomycin A1, 6-cyano-7-nitroquinoxaline-2,3-dione (CNQX), 2,3-dioxo-6-nitro-1,2,3,4-tetrahydrobenzo[f]quinoxaline-7-sulphonamide (NBQX), (*RS*)-α-methyl-4-phosphonophenylglycine (MPPG), 3-((2-methyl-1,3-thiazol-4-yl) ethynyl) pyridine hydrochloride (MTEP), 7-(hydroxyimino) cyclopropa[b] chromen-1a-carboxylate ethyl ester (CPCCOEt), butyl β-carboline-3-carboxylate (β-CCB), 1-(2-[([diphenylmethylene]imino)-oxy]ethyl)-1,2,5,6-tetrahydro-3-pyridinecarboxylic acid hydrochloride (NO711) and 1-[2-[*tris*(4-methoxyphenyl)methoxy]ethyl]-(*S*)-3-piperidinecarboxylic acid (SNAP5114) were obtained from Tocris (Bristol, UK); TTX and 2-(3-carboxypropyl)-3-amino-6-(4 methoxyphenyl)pyridazinium bromide (SR95531) were obtained from Ascent (Weston-super-Mare, UK); kynurenic acid was obtained from Sigma-Aldrich (Poole, Dorset, UK).

### Electrophysiological recordings

The recording chamber and manipulators were mounted on a moveable bridge (Luigs and Neumann, Ratingen, Germany). Slices were visualized by DIC using a Nikon FN1 microscope with a × 40 0.8 NA water dipping lens. Patch-clamp recordings of SOCs were made at room temperature (20 – 24°C), or at 33 ± 1 °C where indicated, using pipettes (2–4 MΩ) containing a high Cl^−^ internal solution of composition (in mm): KCl, 125; NaCl, 10; MgCl_2_, 2; CaCl_2_, 1; HEPES, 10; EGTA, 10; Na_2_ATP, 4; GTP, 0.3. For experiments in δ KO and WT mice, pipettes contained (in mm): CsCl, 130; MgCl_2_, 2; Mg-ATP, 4; Na-GTP, 0.3; HEPES, 10; EGTA, 0.1. To block possible contamination by *N*-methyl-d-aspartate (NMDA) receptor-mediated SICs, either kynurenic acid (3 mm) was included in the ACSF, or MK801 (1 mm) was included in the patch solution (Berretta & Jones, 1996). Current traces from such recordings are displayed inverted to maintain the convention of GABAergic SOCs being outward. Experiments aimed at concomitantly recording both SICs and SOCs and investigating the physiological impact of SOC-mediated hyperpolarizations in current-clamp mode were conducted using intracellular solution containing (in mm): KMeSO_4_, 120; KCl, 5; HEPES, 10; EGTA, 0.1; Na_2_ATP, 4; GTP, 0.5. Intracellular Cl concentration (5 mm) was included to set physiological E_Cl_ in these cells (Ulrich & Huguenard, 1997). Tonic current was measured by rapidly introducing the GABA_A_ antagonist SR95531 to the recording chamber and measuring the resultant change in holding current. Synaptic stimulation was achieved with a computer-controlled constant current-isolated stimulator (STG1002; Multichannel Systems, Germany) and bipolar electrodes placed on the internal capsule. Neuronal currents were recorded using a Multiclamp 700B amplifier, digitized with a Digidata 1440A, and acquired and analysed using pClamp software (Molecular Devices, CA, USA). IPSCs and SOCs were analysed using the Event Detection protocols in Clampfit. Events were classed as SOCs if their amplitude was > 20 pA and their time to peak was > 20 ms. Data were exported to Sigmaplot (Jandel) for further analysis and plotting.

### Statistics

All quantitative data in the text and figures are presented as mean ± SEM. Significance was calculated using unpaired or paired Student's *t*-test as appropriate. Kolmogorov–Smirnov tests were used for population distribution comparisons. Linear correlations were tested using Spearman's Rank correlation. The statistical significance in figures is presented as *P< 0.05, **P< 0.01, ***P< 0.005.

## Results

### Astrocytic GABA release generates SOCs via GABA_A_ receptors

**Fig. 1:**
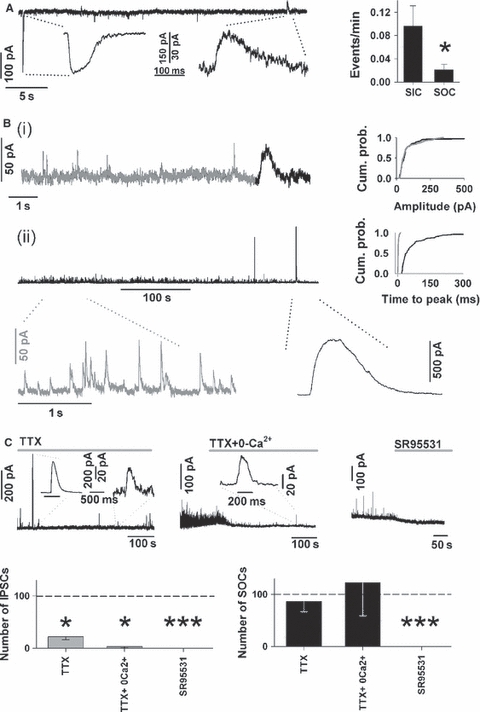
TC neurons of the VB thalamus exhibit fast and slow transient GABA_A_ receptor-mediated events. (A) Current trace displaying concomitant slow inward currents (SICs) and slow outward currents (SOCs) in the same TC neuron. Representative SICs and SOCs are enlarged below. The bar graph shows the relative frequency of SICs and SOCs in neurons examined. (B) (i) Current recording of spontaneous inhibitory postsynaptic currents (IPSCs) and SOCs from a TC neuron recorded using pipettes containing 125 mm KCl. IPSPs can be seen during the grey portion of the trace, whilst a longer duration SOC is shown in black. (ii) Recording from another cell, with expanded traces below displaying fast IPSCs (grey) and a much slower, and unusually large-amplitude, SOC (black). Cumulative probability distributions of amplitude (upper) and time to peak (lower) for IPSCs (grey lines) and SOCs (black lines) are displayed on the right. (C) Traces from experiments displaying spontaneously recorded events in the presence of tetrodotoxin (TTX; 1 μm, left), TTX + 0 mm extracellular Ca^2+^ (middle) and the GABA_A_ antagonist SR95531 (25 μm, right). The bar graphs below show IPSC frequency (grey columns, left) and SOC frequency (black columns, right) normalized to control for each experimental condition. *indicates *P* < 0.05; *** indicates *P* < 0.005.

Patch-clamp recordings were made from TC neurons in the VB thalamus at ages P10–23. In addition to previously described spontaneous SICs (sSICs), corresponding to the activation of NMDA receptors by astrocytically derived glutamate (Fig. 1A; Parri *et al.*, 2001), in some experiments (25 out of 42 recordings) we were able to concomitantly record spontaneous, transient outward currents that had very slow rise times and durations of hundreds of milliseconds (Fig. 1A). Because of their similarity to the SOCs previously described in the olfactory bulb (Kozlov *et al.*, 2006), we adopted the nomenclature of Kozlov *et al.* to identify them. The observed incidence of spontaneous SOCs (sSOCs; 0.02 ± 0.01 SOCs/min) was significantly lower than that of sSICs (0.10 ± 0.03 SICs/min; *P*< 0.05; Fig. 1A).

To focus on these outward inhibitory currents, subsequent experiments were performed using pipettes containing high Cl^−^ in order to isolate and enhance the detection of Cl^−^-mediated currents (see Materials and methods). Under such conditions, spontaneous IPSCs were readily observed, as described previously (Le Feuvre *et al.*, 1997). IPSCs had a mean frequency of 15.60 ± 4.21 Hz (*n* = 5 neurons), a mean time to peak of 2.45 ± 0.14 ms and mean amplitude of 80.70 ± 4.66 pA (*n* = 250 events, pooled from five neurons; Fig. 1B). IPSC frequency was significantly lower in the presence of TTX (1 μm) to block action potential-dependent GABA release (21.96 ± 5.95% of control, *n* = 11 neurons; *P*< 0.05), and following the removal of Ca^2+^ from the ACSF to block voltage-dependent Ca^2+^ entry (3.40 ± 3.00% of control, *n* = 5 neurons; *P*< 0.05; Fig. 1C). Furthermore, application of the selective GABA_A_ receptor antagonist SR95531 (25 μm) abolished all IPSCs (*n* = 4 neurons; Fig. 1C). Thus, conventional synaptic GABA release, presumably from the axon terminals of nRT neurons, underlies the generation of IPSCs in TC neurons of the VB.

In addition to spontaneous IPSCs, sSOCs could also be recorded using pipettes containing high Cl^−^. The mean amplitude of the sSOCs (111.30 ± 31.27 pA, *n* = 52 events) was not significantly different to that of IPSCs, and the distribution of peak amplitudes was also not different (both *P*> 0.05; Fig. 1B). However, the mean time to peak was significantly slower (108.90 ± 34.53 ms, *n* = 52 events; *P*< 0.001) and the distribution of rise times was also significantly different compared with IPSCs (*P*< 0.001; Fig. 1B). In complete contrast to IPSCs, however, the frequency of sSOCs was not affected by application of TTX (control – 0.29 ± 0.16/min; TTX – 0.25 ± 0.06/min, *n* = 9 neurons; *P*> 0.05) or the removal of extracellular Ca^2+^ (122.15 ± 63.56% of control, *n* = 6 neurons; Fig. 1C), indicating a non-synaptic origin. To further investigate the source of GABA responsible for generating SOCs, we treated slices with bafilomycin A1, a depletor of vesicular neuro- and glio-transmitter release. Application of bafilomycin A1 did not significantly affect the frequency of sSOCs (control 0.27 ± 0.09/min, *n* = 19 neurons; bafilomycin 0.38 ± 0.14/min, *n* = 18 neurons; *P*> 0.05) or their amplitude (control 109.60 ± 18.58 pA, *n* = 24 neurons; bafilomycin 137.12 ± 11.34 pA, *n* = 10 neurons; *P*> 0.05), indicating that SOCs are dependent on a non-vesicular GABA release mechanism. sSOC frequency was also not affected by the combined block of ionotropic and metabotropic glutamate receptors following the application of D-AP5 (50 μm), NBQX or CNQX (20 μm), MTEP (10 μm), CPCCOEt (100 μm), LY341495 (2 μm) and MPPG (100 μm; control – 0.24 ± 0.06/min, *n* = 9 neurons; antagonists – 0.21 ± 0.07/min, *n* = 5 neurons). However, like IPSCs, sSOCs were completely abolished by the GABA_A_ receptor antagonist SR95531 (*n* = 5 neurons; Fig. 1C).

**Fig. 2:**
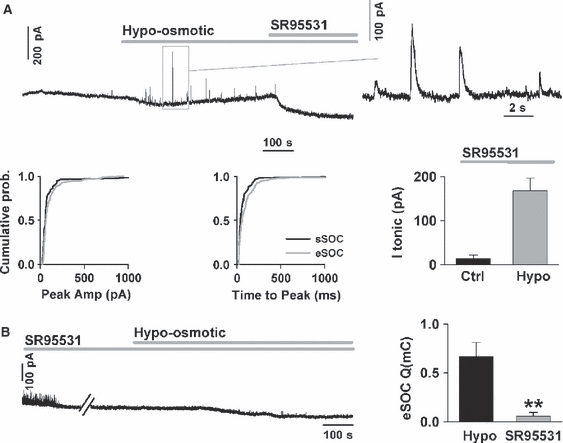
Slow outward currents (SOCs) are evoked by hypo-osmotic stimulus. (A) Current trace from a TC neuron showing the increase in occurrence of SOCs following bath application of hACSF. Several evoked SOCs (eSOCs) are expanded to the right. Note how application of SR95531 (25 μm) not only blocks eSOCs but also causes an inward shift in current, revealing the presence of an outward tonic GABA_A_ current. Cumulative probability distributions below compare the amplitude (left) and time to peak (right) of eSOCs (grey) to spontaneous SOCs (sSOCs; black). On the far right is a bar graph comparing the amplitude of the tonic current under control conditions (Ctrl) and following hypo-osmotic stimulus (Hypo). (B) Current trace showing that hypo-osmotic stimulus fails to induce eSOCs or alter the holding current if SR95531 is applied beforehand. The bar graph on the right shows the effect of SR95531 on eSOC charge (*Q*) compared with hypo-osmotic stimulus alone. ** indicates *P* < 0.001.

The incidence of sSOCs was low, with events only occurring every few minutes. We therefore attempted to increase their frequency by applying a hypo-osmotic stimulus, as described previously in the olfactory bulb (Kozlov *et al.*, 2006). Application of hACSF elicited evoked SOCs (eSOCs) at a frequency of 1.55 ± 0.43/min (*n* = 14 neurons; *P*< 0.001 compared with sSOC frequency; Fig. 2A). The mean rise time and mean amplitude of eSOCs (136.97 ± 19.66 ms and 134.19 ± 13.21 pA, respectively, *n* = 14 neurons) were not significantly different to sSOCs recorded in the same neurons (100.01 ± 19.65 ms and 109.60 ± 18.58 pA, respectively; both *P*> 0.05; Fig. 2A). However, the population distributions for rise times and peak amplitudes were significantly different (*P*< 0.05 and *P*< 0.005, respectively), with the eSOC population containing larger, slower events (Fig. 2A). Bath application of SR95531 following hACSF application not only blocked all eSOCs but also revealed a large tonic GABA_A_ receptor-mediated current compared with normal, control ACSF (control – 14.30 ± 7.06 pA, *n* = 8 neurons; hACSF – 168.10 ± 28.36 pA, *n* = 8 neurons; *P*< 0.005; Fig. 2A). Thus, GABA release caused by hypo-osmotic stimulation not only increases the incidence of SOCs but also contributes to tonic GABA_A_ inhibition. Application of SR95531 before the hypo-osmotic stimulus prevented the generation of eSOCs (control – 0.67 ± 0.14 mC, *n* = 8 neurons; SR95531 – 0.06 ± 0.04 mC, *n* = 5 neurons; *P*< 0.01; Fig. 2B), confirming that eSOCs, like sSOCs, are exclusively mediated by GABA_A_ receptors.

In summary, these results show that SOCs are GABA_A_ receptor-mediated events that have markedly different properties to GABA_A_ receptor-mediated IPSCs, notably occurrence, onset and duration (Figs 1 and 2). In addition, whereas IPSCs are sensitive to TTX, external Ca^2+^ concentration and bafilomycin A1, SOCs are insensitive to all three of these manipulations. Thus, IPSCs arise due to conventional vesicular GABA release from neuron terminals, but SOCs are dependent upon an alternate GABA source that is non-synaptic and non-vesicular. These data, together with the sensitivity of SOCs to hypo-osmotic stimulation, suggest that SOCs are generated in response to the release of GABA from astrocytes, probably through a volume-regulated anion channel mechanism as previously implicated in the olfactory bulb (Kozlov *et al.*, 2006).

### Nucleus-specific distribution and developmental profile of SOCs

**Fig. 3:**
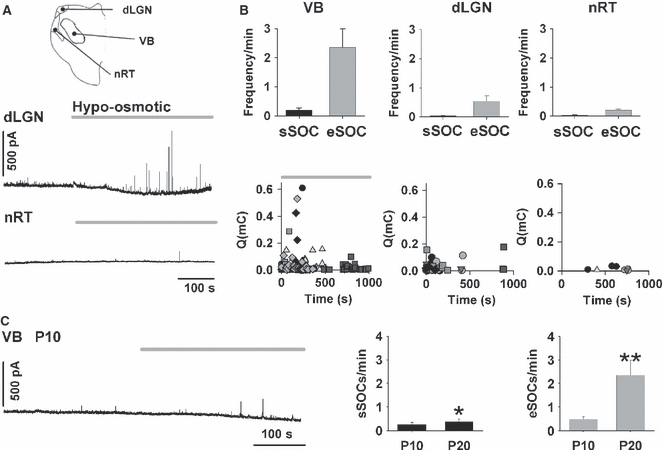
Nucleus-specific and age-dependent expression of slow outward currents (SOCs). (A) Schematic diagram showing the relative locations of the ventrobasal (VB), dorsal lateral geniculate nucleus (dLGN) and nucleus reticularis thalami (nRT) in a slice preparation. Below, example traces of evoked SOCs (eSOCs) following hypo-osmotic stimulus (grey line) in a TC neuron from the dLGN (upper trace), and in a neuron from the nRT (lower trace). (B) Summary bar graphs (upper row) of spontaneous SOC (sSOC; black bars) and eSOC (grey bars) frequencies for the different nuclei. Below are plots of charge (*Q*) carried by eSOCs in the different nuclei. Each symbol represents a single SOC, SOCs from the same cell are coded with the same symbol, time 0 denotes time of start of hypo-osmotic stimulus (grey bar in VB example). (C) Trace showing eSOCs elicited by hypo-osmotic stimulus in a TC neuron recorded in a slice taken from an animal at P10. To the right are bar graphs comparing the frequency of sSOCs (black bars) and eSOCs (grey bars) in TC neurons of the VB at P10 (left) and P20 (right). * indicates *P* < 0.05; ** indicates *P* < 0.01.

To determine the functional expression of SOCs in the thalamus, we compared sSOCs and eSOCs in thalamic nuclei that have different anatomical and functional properties at the same age (P18–23) – the VB nucleus, which receives somatosensory input and is comprised solely of glutamatergic TC neurons (Barbaresi *et al.*, 1986; Harris & Hendrickson, 1987); the dorsal lateral geniculate nucleus (dLGN), which receives visual input and contains a population of GABAergic interneurons in addition to TC neurons (Ohara *et al.*, 1983); and the nRT, a thalamic nucleus comprised solely of GABAergic neurons (Spreafico *et al.*, 1991; Fig. 3A). sSOCs were most prevalent in the VB thalamus (0.20 ± 0.07/min, *n* = 8 neurons), followed by the dLGN (0.03 ± 0.02/min, *n* = 9 neurons) and then the nRT (0.02 ± 0.02/min, *n* = 4 neurons; Fig. 2A and B). eSOCs also followed the same pattern, being most common in the VB (2.35 ± 0.63/min, *n* = 8 neurons), followed by the dLGN (0.53 ± 0.18/min, *n* = 9 neurons) and then the nRT (0.20 ± 0.04/min, *n* = 5 neurons; Fig. 3A and B). These data suggest that the VB has the greatest potential for non-synaptic astrocytic GABA signalling, but also the greatest functional impact, as the total charge carried by eSOCs following hACSF application in the VB thalamus was significantly greater than in both the dLGN and the nRT (VB – 0.67 ± 0.14 mC, *n* = 8 neurons; dLGN – 0.19 ± 0.06 mC, *n* = 7 neurons; nRT – 0.03 ± 0.02 mC, *n* = 4 neurons; VB vs. dLGN, *P*< 0.01; VB vs. nRT, *P*< 0.01; Fig. 3B).

We also investigated sSOC frequency at different ages in TC neurons of the VB. sSOC frequency was significantly lower in TC neurons at P10 compared with P20 (P10 – 0.30 ± 0.01/min, *n* = 19 neurons; P20 – 0.40 ± 0.05/min, *n* = 11 neurons; *P*< 0.01; Fig. 3C). eSOC frequency in response to hypo-osmotic stimulation was similar to sSOC frequency in neurons from P10 animals (0.49 ± 0.10/min, *n* = 6 neurons), whereas the incidence of eSOCs was greater than sSOCs in neurons from P20 animals (2.35 ± 0.63/min, *n* = 8 neurons). eSOC frequency was significantly different between the two ages (*P*< 0.01; Fig. 3B). The occurrence of SOCs therefore exhibits both a nucleus-specific distribution, being more prevalent in the VB than both the dLGN and nRT, and a developmental emergence in the VB.

### SOCs are generated by δ-subunit-containing GABA_A_ receptors

**Fig. 4:**
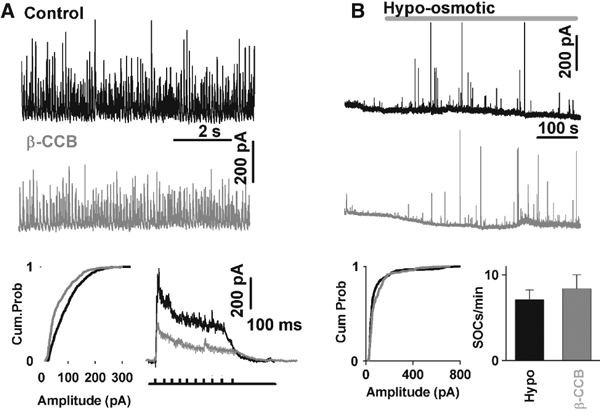
Slow outward currents (SOCs) are not mediated by synaptic γ2-subunit-containing receptors. (A) Current traces showing IPSCs under control conditions (black) and following β-CCB (1 μm) application (grey) in the same neuron. Below is a cumulative probability plot (left) of IPSC amplitude in the absence (black line) and presence (grey line) of β-CCB. On the right are traces showing the effect of β-CCB (grey) on evoked IPSCs elicited by stimulation of the nRT, compared with control (black). (B) Current traces showing the effects of hypo-osmotic stimulus in the absence (black trace) and presence (grey trace) of β-CCB. Below is a cumulative distribution plot (left) of eSOC amplitude in the absence (black line) and presence (grey line) of β-CCB. The bar graph on the right shows the frequency of eSOCs in the two experimental conditions.

**Fig. 5:**
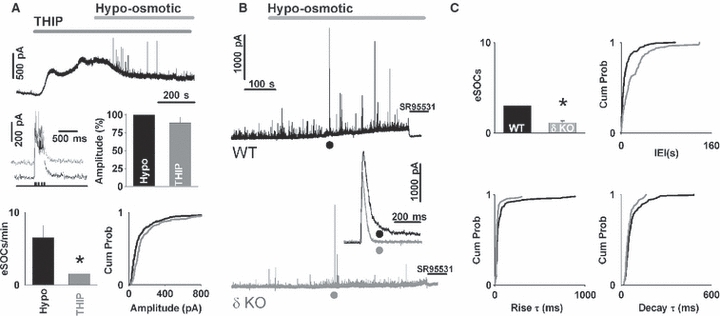
Slow outward currents (SOCs) are mediated by extrasynaptic synaptic δ-subunit-containing receptors. (A) Example traces of evoked SOCs (eSOCs) generated by hACSF application following THIP (1 μm) exposure. Below are traces showing evoked IPSCs from the neuron before (black trace) and after (grey trace) THIP application. To the right is a bar graph comparing evoked IPSC amplitude under control conditions (black column) and in the presence of THIP (grey column) normalized to control. At the bottom is a bar graph (left) comparing eSOC frequency in the absence (black column) and presence (grey column) of THIP. To the right is a cumulative probability plot of eSOC amplitude prior to (black line) and after (grey line) THIP application. (B) Current trace showing SOCs evoked by hypo-osmotic stimulus in a VB TC neuron recorded from a wild-type (WT) mouse (top), and a δ-subunit knockout (δ KO) littermate (bottom, grey trace). Inset shows expanded overlaid traces of the eSOCs indicated in the main traces. (C) Bar graph showing the frequency of eSOCs in WT (black column) and δ KO (grey column) mice. To the right and below are cumulative probability distribution plots comparing the inter-event intervals (IEIs), rise time constant and decay time constant for δ KO (grey lines) and WT mice (black lines). * indicates *P* < 0.05.

TC neurons of the VB contain relatively few GABA_A_ receptor subtypes. Synaptic receptors are comprised of α1β2γ2-subunits and are responsible for the generation of IPSCs, whereas α4β2δ receptors are present in the extrasynaptic membrane and generate tonic currents (Wong & Snead, 2001; Belelli *et al.*, 2005; Jia *et al.*, 2005; Chandra *et al.*, 2006; Peden *et al.*, 2008; Herd *et al.*, 2009). Both the nucleus-specific occurrence and developmental profile of SOCs mirrors the nucleus-specific distribution and postnatal development of the δ-subunit, i.e. the δ-subunit is strongly expressed in the VB and dLGN but is absent from the nRT (Pirker *et al.*, 2000), and postnatally the δ-subunit is only present from ∼P12 onward in rats (Laurie *et al.*, 1992). Because astrocytic glutamate release has been suggested to target extrasynaptic receptors (Halassa *et al.*, 2007), we therefore postulated that SOCs are generated by the activation of δ-subunit-containing extrasynaptic receptors, as opposed to γ2-subunit-containing synaptic receptors. To test the role of different GABA_A_ receptor populations in the generation of SOCs we used the benzodiazepine site inverse agonist β-CCB to target synaptic receptors, and the δ-subunit-selective agonist THIP to target extrasynaptic receptors. Application of β-CCB (1 μm, *n* = 4 neurons) caused a significant reduction in IPSC amplitude (control – 93.90 ± 2.25 pA; β-CCB – 69.40 ± 2.17 pA, 608 and 533 events, respectively; *P*< 0.005; Fig. 4A). Evoked IPSCs generated by stimulation of the nRT were also significantly reduced by application of β-CCB (control – 493.62 ± 101.87 pA; β-CCB – 282.40 ± 94.92 pA, *n* = 6 neurons; *P*< 0.05; Fig. 4A). Analysis of the effect of β-CCB on eSOCs (*n* = 4 neurons) showed a small, but significant, increase in amplitude (control – 89.70 ± 9.11 pA; β-CCB – 98.20 ± 10.30 pA, 188 and 204 events, respectively; *P*< 0.05), but no effect on frequency (control – 7.11 ± 1.11/min; β-CCB – 8.37 ± 1.63/min; Fig. 4B). Thus, IPSCs are indeed generated by synaptic, γ2-subunit-containing receptors, but SOCs are not dependent on this receptor population. Application of the δ-subunit-selective agonist THIP (1 μm) resulted in a large tonic GABA_A_ current (285.96 ± 63.12 pA, *n* = 9 neurons), as described previously (Belelli *et al.*, 2005; Jia *et al.*, 2005; Cope *et al.*, 2009; Fig. 5A). Following hACSF application in the presence of THIP, eSOCs occurred at a reduced frequency (hACSF – 6.54 ± 1.72/min, *n* = 7 neurons; hACSF + THIP – 1.56 ± 0.73/min, *n* = 8 neurons; *P*< 0.05; Fig. 5A). In addition, the peak amplitude of eSOCs was significantly increased in the presence of THIP (hACSF – 165.04 ± 16.20 pA, *n* = 286 events from seven neurons; hACSF + THIP – 214.98 ± 30.32 pA, *n* = 83 events from eight neurons; *P*< 0.005). By comparison, the amplitude of the evoked IPSCs elicited by stimulation of the nRT was not significantly affected by THIP (88.32 ± 8.10% of control, *n* = 5 neurons; Fig. 5A), indicating that the reduction in eSOC frequency was not due to a simple shunting of the membrane.

To further probe the role of δ-subunit-containing receptors we compared eSOCs in δ KO mice and WT littermates. In WT mice, hACSF application elicited eSOCs similar to those seen in rat VB TC neurons (3.01 ± 0.84 eSOCs/min, *n* = 4 neurons; Fig. 5B). The number of eSOCs was significantly reduced in mice lacking the δ-subunit (1.12 ± 0.49 eSOCs/min, *n* = 5 neurons, *P*< 0.05; Fig. 5B), underlined also by the significant shift in the cumulative probability distribution for inter-event intervals (WT – 8.94 ± 1.12 s, *n* = 150; δ KO – 22.86 ± 3.19 s, *n* = 83, *P*< 0.0001; Fig. 5C). In addition, the kinetics of eSOCs were significantly different between the δ KO and WT mice (WT τ_rise_– 66.27 ± 11.16 ms, *n* = 151; δ KO τ_rise_– 29.43 ± 4.74 ms, *n* = 84, *P*< 0.001; WT τ_decay_– 74.19 ± 5.63 ms; δ KO τ_decay_– 53.68 ± 3.63 ms, *P*< 0.05; Fig. 5C).

Together, these data show that SOCs are generated by the activation of extrasynaptic δ-subunit-containing GABA_A_ receptors, as they are: (i) insensitive to the benzodiazepine site inverse agonist β-CCB, which specifically targets γ2-subunit-containing synaptic receptors; (ii) sensitive to THIP, a preferential agonist at δ-subunit-containing receptors; and (iii) less frequent in mice following the genetic ablation of the δ-subunit.

### The anti-epileptic vigabatrin augments SOCs

**Fig. 6:**
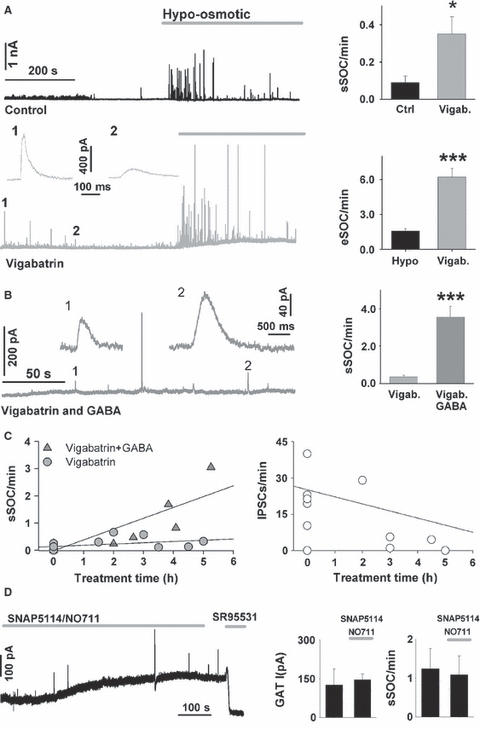
Slow outward currents (SOCs) are augmented by the anti-epileptic vigabatrin. (A) Current traces from TC neurons showing spontaneous activity and response to a hypo-osmotic stimulus under control conditions (black trace), and in slices pre-treated with 200 μm vigabatrin (grey trace). Expanded spontaneous SOCs (sSOCs) are as indicated (1 and 2). The bar graphs on the right illustrate the increase in frequency of sSOCs (top) and evoked SOCs (eSOCs; bottom) following pre-treatment with vigabatrin. (B) Current trace from a TC neuron showing sSOCs following incubation with 200 μm vigabatrin and 200 μmγ-aminobutyric acid (GABA). Expanded sSOCs are as indicated (1 and 2). To the right is a bar graph comparing the effect of vigabatrin + GABA pre-treatment (dark grey column) on sSOC frequency compared with vigabatrin pre-treatment alone (grey column). (C) The plot on the left illustrates the time dependence of vigabatrin pre-treatment on sSOC frequency. Circles show sSOC frequency for different experiments in slices pre-treated with vigabatrin alone, triangles show experiments from slices pre-treated with vigabatrin + GABA. The plot on the right shows the time-dependent effect of vigabatrin pre-treatment on inhibitory postsynaptic currents (IPSCs). Plots are fitted with linear regression lines. (D) Current trace showing the effect of co-application of NO711 and SNAP5114 (20 μm and 40 μm, respectively) on holding current and sSOC frequency in a TC neuron from a vigabatrin-pre-treated slice. Application of SR95531 (25 μm) abolishes the transporter blocker-induced increase in tonic GABA_A_ current. The bar graphs on right show the effect of GABA transporter (GAT) blockers on tonic current amplitude (left) and sSOC frequency (right) in the presence of vigabatrin and GABA alone, and together with NO711 and SNAP5114. * indicates *P* < 0.05; *** indicates *P* < 0.005.

The lack of interneurons in the VB thalamus, the distinct properties of SOCs and IPSCs, and the evoking of SOCs by hypo-osmotic stimuli that are known to induce astrocytic transmitter release (Kimelberg *et al.*, 2006), along with a previous report showing that SOCs in the olfactory bulb are dependent on astrocytic GABA release (Kozlov *et al.*, 2006), all point to SOCs in TC neurons of the VB thalamus being generated by GABA released from astrocytes. Because GABA uptake transporters in the VB thalamus are only present on astrocytes (Barbaresi *et al.*, 1986; De Biasi *et al.*, 1998), we hypothesized that inhibiting the catabolism of GABA by blocking the action of GABA transaminase (GABA-T) should increase astrocytic GABA levels and lead to an increase in SOCs, if they are indeed astrocytically derived. To test this hypothesis we used the GABA-T inhibitor and anti-epileptic vigabatrin. Treatment of slices with vigabatrin (200 μm) significantly increased both the frequency of sSOCs (control – 0.09 ± 0.035/min, *n* = 7 neurons; vigabatrin 0.35 ± 0.09/min, *n* = 6 neurons; *P*< 0.05) and the frequency of eSOCs (control – 2.95 ± 0.64/min, *n* = 7 neurons; vigabatrin 6.21 ± 0.75/min, *n* = 5 neurons; *P*< 0.005; Fig. 6A). In addition, the time to peak (control – 44.15 ± 3.40 ms, *n* = 102 events from seven neurons; vigabatrin 85.69 ± 14.33 ms, *n* = 194 events from five neurons; *P*< 0.001), but not the peak amplitude (control 184.34 ± 24.37 pA; vigabatrin 228.76 ± 24.17 pA; *P*> 0.05), of eSOCs was also significantly affected. There was also a trend in the frequency of both sSOCs and eSOCs to increase in a vigabatrin-treated time-dependent manner (sSOCs –*R* = 0.52, *P*= 0.07; eSOCs –*R* = 0.81, *P*< 0.005; Fig. 6C). Conversely, as expected if the astrocyte–neuronal GABA cycle was perturbed, the frequency of IPSCs declined in a time-dependent manner (*R* = −0.88, *P*< 0.01), as previously described in dentate gyrus granule cells (Overstreet & Westbrook, 2001), further highlighting the different sources of GABA mediating IPSCs and SOCs (Fig. 6C).

We further probed the role of astrocytic GABA and GABA-T in the generation of SOCs by testing the hypothesis that promoting GABA uptake by increasing the extracellular GABA concentration should lead to a build-up of astrocytic GABA, so that there is more available for release. Slices were treated with 200 μm GABA in conjunction with 200 μm vigabatrin, and the frequency of sSOCs in treated slices was measured at varying time points. Increasing available GABA not only increased sSOC frequency (vigabatrin – 0.35 ± 0.09/min, *n* = 6 neurons; vigabatrin + GABA – 3.56 ± 0.56/min, *n* = 5 neurons; *P*< 0.005), but also the time-dependent correlation of sSOCs with vigabatrin treatment (*R* = 0.86, *P*< 0.001; Fig. 6B and C). Previous studies with vigabatrin (Overstreet & Westbrook, 2001; Wu *et al.*, 2001, 2003) have suggested that increased intracellular GABA leads to reversal of GATs. We tested whether vigabatrin increased SOCs via reversal of GATs by applying the GAT-1- and GAT-3-selective blockers NO711 and SNAP5114. Co-application of NO711 (20 μm) and SNAP5114 (40 μm) increased the tonic GABA_A_ current, consistent with the block of GABA uptake, but had no effect on the increase in frequency of sSOCs caused by treatment with vigabatrin and GABA (vigabatrin + GABA – 1.25 ± 0.51/min; NO711, SNAP5114, vigabatrin + GABA – 1.09 ± 0.47/min, *n* = 5 neurons; *P*> 0.05; Fig. 6D). These data show that the increase in SOC frequency following vigabatrin treatment is not due to reversal of GATs, and that GABA uptake is unaffected as the block of GATs leads to an increase in the tonic GABA_A_ current, not a reduction as would be expected if transporters were reversed.

### Slow GABAergic hyperpolarizations

To determine the physiological manifestation and impact of SOCs, we recorded from VB TC neurons in current-clamp mode. Under control conditions we recorded two types of inhibitory activity: inhibitory postsynaptic potentials (IPSPs) and spindle waves. Both these have been previously described and shown to be derived from synaptic afferent activity emanating from the nRT onto VB TC neurons (Gentet & Ulrich, 2003).

**Fig. 7:**
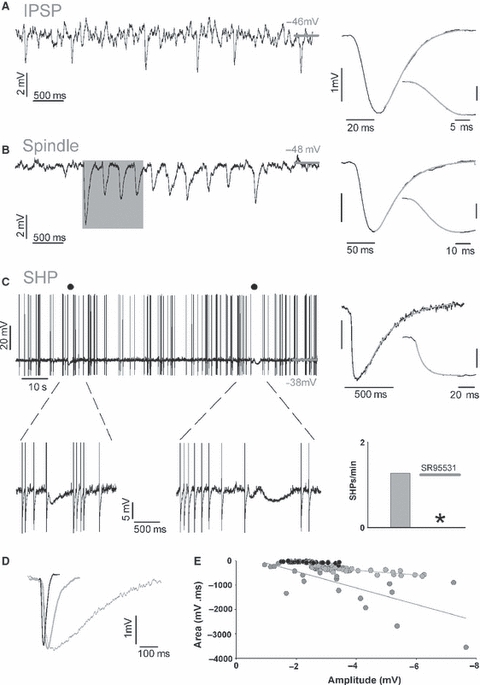
GABAergic slow hyperpolarizing potentials (SHPs). (A) Current-clamp recording from a VB TC neuron illustrating typical spontaneous fast inhibitory postsynaptic potentials (IPSPs). On the right is an averaged trace of IPSPs. Event decay is fitted with a product of two exponentials (grey line). Inset illustrates expanded rising phase of the mean IPSP fitted with a product of two exponentials (grey line). (B) Recording in current-clamp of spindle oscillations recorded in a TC neuron. Grey block denotes 1-s timeframe, illustrating stereotypical 4-Hz pattern. On the right is an averaged trace of spindle hyperpolarizations. Event decay is fitted with a product of two exponentials (grey line). Inset illustrates expanded rising phase of the mean hyperpolarization fitted with a product of two exponentials (grey line). (C) Current-clamp recording from a VB TC neuron following treatment with vigabatrin (200 μm). The highlighted SHPs are enlarged below. The average trace of SHPs is shown to the right. Event decay is fitted with a single exponential (grey line). Inset illustrates the expanded rising phase of the mean SHP fitted with a single exponential (grey line). The bar graph below shows the block of SHPs following the application of SR95531. (D) Superimposed average IPSPs (black trace), spindle hyperpolarizations (grey trace) and SHPs (dark grey trace). (E) Plots of inhibitory event area against event amplitude. IPSPs (black), spindle hyperpolarizations (grey) and SHPs (dark grey), fitted with corresponding linear regression plots. Scale bar for traces, as illustrated. Current was injected to set *V*_m_ to depolarized ‘tonic’ mode potentials. The stated *V*_m_ in current-clamp traces is indicated by a dark grey bar. The amplitude bar for all fitted traces is 1 mV. * indicates *P* < 0.05.

IPSPs were seen as spontaneous transient events, caused a −2.3 ± 0.06 mV (*n* = 56) hyperpolarization and had a time to peak of 26.69 ± 0.51 ms. A mean IPSP averaged from 56 IPSPs had rise and decay fitted by a product of two exponentials (corr = 0.98 and 0.89, respectively; Fig. 7A). Such IPSPs correspond to VB thalamus GABA release elicited by single nRT action potentials (APs)(Gentet & Ulrich, 2003). However, nRT neurons also produce VB inhibitory hyperpolarizations by volleys of APs generated during nRT neuron bursting. Such recurrent bursts occur during spindles, which are 3–4 Hz oscillatory activity generated by intrathalamic networks between the inhibitory nRT neurons and excitatory TC neurons. We therefore analysed the inhibitory hyperpolarizing events occurring during spindles in VB TC neurons as a way of identifying inhibitory events generated as the result of nRT AP bursts.

Hyperpolarizations had an amplitude of −3.57 ± 0.16 mV (*n* = 46) and time to peak of 54 ± 0.93 ms. A mean spindle hyperpolarization averaged from 46 events had rise (correlation 0.98) and decay fitted by products of two exponentials (corr = 0.93; Fig. 7B).

To detect hyperpolarizations induced by SOCs we recorded in current-clamp in slices at 33 ºC that had been treated with 200 μm vigabatrin to increase sSOC frequency. In these recordings we observed slow hyperpolarizing potentials (SHPs) that hyperpolarized the membrane potential away from action potential threshold (Fig. 7C). SHPs caused a −3.21 ± 0.34 mV (*n* = 27) hyperpolarization and had a time to peak of 129.61 ± 12.50 ms. A ‘mean’ SHP averaged from 27 SHPs recorded from five cells had rise and decay time constants that were fitted by single exponentials (τ_rise_ = 10.11 ms, corr = 1.00; τ_decay_ = 300 ms, corr = 0.92; Fig. 7B), supporting the notion that SHPs are single events rather than compound bursts of synaptic activity. As expected if SHPs were generated by SOCs, application of SR95531 (20 μm) blocked all SHPs (Fig. 7C). SOCs are therefore capable of modulating thalamic output through the generation of SHPs that reduce TC neuron excitability. The slower kinetics and the fitting of the rise time with a single exponential therefore distinguish SHPs from spindle hyperpolarizations and IPSPs (Fig. 7D). The relationship between amplitude and area was steepest for SHPs (Fig. 7D; *R*: IPSP = 0.69, spindle = 0.85, SHP = 0.71, *P* < 0.001).

## Discussion

### Source of GABAergic SOCs

The comparatively simple anatomical and synaptic structure of the rodent VB thalamus, combined with the conspicuous lack of GABAergic interneurons, make it an attractive model for investigating astrocytic function and, in particular, gliotransmitter release. Our data from TC neurons show that SOCs are dependent on astrocytic GABA release. Firstly, SOCs, unlike IPSCs, are not dependent on action potentials, Ca^2+^ entry or vesicular GABA release, indicating that they are non-synaptically derived. Although alternate non-synaptic neuronal GABA release mechanisms have been demonstrated (Koch & Magnusson, 2009), and in the dLGN GABA can be released from interneuron dendrites (Acuna-Goycolea *et al.*, 2008), the absence of interneurons in the VB thalamus, coupled to the fact that dendritic GABA release is also vesicular and dependent on extracellular Ca^2+^, precludes this mechanism in accounting for our observations (Isaacson, 2001). Secondly, SOCs are induced by hypo-osmotic stimuli that are an established method for inducing astrocytic amino acid release (Kimelberg *et al.*, 2006), and the method employed in the olfactory bulb to elicit astrocytically mediated SOCs. Finally, selectively targeting astrocyte GABA cycling by promoting GABA uptake by GATs and inhibiting GABA catabolism increases the incidence of SOCs but concomitantly decreases IPSC frequency, providing further evidence that SOCs are astrocytically derived. The specific mechanism involved in astrocytic GABA release remains to be identified, but the most likely candidate is a volume-regulated anion channel, as previously implicated in the olfactory bulb (Kozlov *et al.*, 2006). Another candidate may be the recently described bestrophin channel (Lee *et al.*, 2010). However, our data do demonstrate that reversed GABA transport is not responsible for astrocytic GABA release, even in conditions where astrocytic GABA levels are elevated following block of GABA-T with vigabatrin or increased ambient GABA. This is in contrast to previous findings where reversed GABA transport has been observed under physiologically relevant conditions (Wu *et al.*, 2007), and in response to vigabatrin treatment (Wu *et al.*, 2001, 2003), although this is not a universal finding (Overstreet & Westbrook, 2001).

### Astrocytic GABA release targets of δ-subunit-containing GABA_A_ receptors

In addition to fast IPSCs and persistent tonic currents, TC neurons therefore exhibit a third type of GABA_A_ receptor-mediated inhibition of intermediate duration, similar, but even slower, to the GABA_A,slow_ IPSCs previously described in hippocampal and cortical pyramidal neurons that are mediated by an unusual GABAergic interneuron, the neurogliaform cell (Banks *et al.*, 1998; Szabadics *et al.*, 2007; Olah *et al.*, 2009). In the thalamus, astrocytically released GABA appears to target δ-subunit-containing extrasynaptic GABA_A_ receptors, rather than γ2-subunit-containing synaptic receptors, as SOCs were insensitive to β-CCB, a benzodiazepine site, i.e. γ2-subunit-specific, inverse agonist, but were sensitive to modulation by the δ-subunit-selective agonist THIP, and were less frequent in mice lacking the δ-subunit. However, SOCs were not completely abolished in δ KO mice, but this is not surprising as extrasynaptic GABA_A_ receptors of unknown composition are present in TC neurons of δ KO mice and generate a residual tonic current (Cope *et al.*, 2009; Herd *et al.*, 2009). Indeed, the subunit composition of this unknown receptor subtype may explain the difference in SOC kinetics between δ KO and WT mice. To the best of our knowledge, therefore, this is the first demonstration that extrasynaptic GABA_A_ receptors are more than simple detectors of ambient GABA, but can mediate distinct, transient events in response to GABA release, whatever the source. Furthermore, tonic current amplitude increased substantially in response to hypo-osmotically induced astrocytic GABA release, and there is every reason therefore to believe that, despite the relatively low incidence of sSOCs, astrocytes are a source of GABA for tonic GABA_A_ currents under physiologically relevant conditions.

We suggest that the THIP-induced decrease in SOC frequency, but increase in amplitude, is due to the fact that THIP is a full agonist at δ-subunit-containing receptors, whereas GABA is only a partial agonist (Brown *et al.*, 2002; Storustovu & Ebert, 2006). The persistent presence of THIP fully occupies the extrasynaptic receptors, not only generating a large tonic current, but also preventing the binding of GABA to the receptors for most of the time. Thus, THIP causes a decrease in SOC frequency. On rare occasions, however, enough GABA is released to displace THIP from the receptors, thereby enabling the generation of SOCs. Because a substantial quantity of GABA must be required to do this, only large events are observed, and so there is a bias toward larger events in the presence of THIP. In short, THIP competes against the endogenous agonist for occupancy of the receptors, and GABA only succeeds in activating the receptor when a suitably high concentration is released.

### SOCs and GABA-T

The exacerbation of SOCs by vigabatrin highlights a potentially novel therapeutic action for this anti-epileptic drug. Indeed, the concentration used in our study (200 μm) is within the plasma concentration range seen in epileptic patients (132–518 μm;Sanchez-Alcaraz *et al.*, 1996). Vigabatrin remains a frontline treatment for a variety of seizure types (Wheless *et al.*, 2007), where increased GABAergic inhibition may be beneficial. However, vigabatrin treatment is contraindicated for typical absence epilepsy, the major form of epilepsy associated with cortico-TC networks. Indeed, vigabatrin has been shown to exacerbate seizures in human absence epilepsy (Lortie *et al.*, 1993; Parker *et al.*, 1998; Perucca *et al.*, 1998), and in rodent models that exhibit spike-and-wave discharges, the clinical hallmark of absence seizures (Vergnes *et al.*, 1984; Hosford & Wang, 1997; Bouwman *et al.*, 2007). These observations are consistent with the finding that extrasynaptic GABA_A_ receptor gain-of-function in the thalamus is sufficient to induce absence seizures (Cope *et al.*, 2009), and that administration of THIP can initiate spike-and-wave discharges (Fariello & Golden, 1987; Cope *et al.*, 2009). Because astrocytic GABA release also targets extrasynaptic receptors, SOCs may play an important pathological role in the appearance of absence seizures.

### Physiological roles of astrocytic GABA release

Whilst synaptic and, to a lesser extent, extrasynaptic GABA signalling in the thalamus has been extensively studied, the physiological roles of astrocytic GABA release in the thalamus are unclear. In our study, we demonstrate that SOCs can occur spontaneously and have a physiological impact, generating SHPs that reduce TC neuron excitability through membrane hyperpolarization, and probably membrane shunting. SOCs therefore have the potential to shape TC neuron output.

It is noteworthy that the incidence of SOCs in thalamic nuclei is inversely proportional to the presence of GABAergic neurons, with SOCs more prevalent in the VB thalamus, a nucleus devoid of thalamic interneurons, compared with the dLGN, and rarely occurring in the nRT, which contains only GABAergic neurons. Despite the apparent low frequency of spontaneously occurring SOCs, which might suggest that they are not physiologically relevant, their increased incidence with age, and capacity to be upregulated by GABA supply indicates that they may also be upregulated in some physiological or pathophysiological conditions. Indeed by analogy with other gliotransmitters (Parri *et al.*, 2001; Henneberger *et al.*, 2010), it may also be expected that certain neurotransmitter systems act to elicit astrocytic GABA release. Astrocytes may therefore function as inhibitory cellular elements, and some of the roles of thalamic GABAergic neurons may be adopted by astrocytes in the VB thalamus. In this context, astrocytic glutamate release, which generates SICs in the thalamus (Parri *et al.*, 2001), has been shown to participate in local, synchronized excitation of neurons in the hippocampus (Angulo *et al.*, 2004; Fellin *et al.*, 2004) and nucleus accumbens (D'Ascenzo *et al.*, 2007), probably via extrasynaptic receptors. SOCs may therefore be a mechanism for local, synchronized inhibition of neurons as astrocytic GABA release would be expected to affect multiple neurons within their domain (Halassa *et al.*, 2007). Furthermore, abnormal astrocytic glutamate release may underlie neuronal hypersynchronization (Tian *et al.*, 2005), and therefore aberrant astrocytic GABA release may also be pathological. Indeed the physiological roles of extrasynaptic receptors are becoming increasingly apparent, and aberrant extrasynaptic receptor function occurs in several pathophysiological conditions (Maguire *et al.*, 2005; Naylor *et al.*, 2005; Scimemi *et al.*, 2005; Cope *et al.*, 2009; Pavlov *et al.*, 2009; Rothman *et al.*, 2009). However, the contribution of astrocytic GABA signalling to these physiological and pathological behaviours has yet to be determined.

In conclusion, our study describes a previously unrecognized form of astrocyte–neuron GABA signalling in the thalamus that specifically targets extrasynaptic GABA_A_ receptors. Whilst the physiological roles of this signalling remain to be elucidated, its augmentation by vigabatrin suggests that astrocytic GABA release may be a therapeutic target for some anti-epileptics, further highlighting the importance of glia in both physiological and pathophysiological processes in the CNS.

## Abbreviations

ACSF: artificial cerebrospinal fluid
AP: action potential
dLGN: dorsal lateral geniculate nucleus
eSOC: evoked slow outward current
GABA: γ-aminobutyric acid
GABA-T: GABA transaminase
GAT: GABA transporter
hACSF: hypo-osmotic artificial cerebrospinal fluid
IPSC: inhibitory postsynaptic current
IPSP: inhibitory postsynaptic potential
KO: knockout
NMDA: *N*-methyl-d-aspartate
nRT: nucleus reticularis thalami
SHP: slow hyperpolarizing potential
SIC: slow inward current
SOC: slow outward current
sSIC: spontaneous slow inward current
sSOC: spontaneous slow outward current
TC: thalamocortical
TTX: tetrodotoxin
VB: ventrobasal
WT: wild-type
